# Elemental Composition and Cell Mass Quantification of Cultured Thraustochytrids Unveil Their Large Contribution to Marine Carbon Pool

**DOI:** 10.3390/md19090493

**Published:** 2021-08-29

**Authors:** Biswarup Sen, Jiaqian Li, Lyu Lu, Mohan Bai, Yaodong He, Guangyi Wang

**Affiliations:** 1 Center for Marine Environmental Ecology, School of Environmental Science and Engineering, Tianjin University, Tianjin 300072, China; bsen@tju.edu.cn (B.S.); lijiaqian@tju.edu.cn (J.L.); lvluu@tju.edu.cn (L.L.); bmh@zju.edu.cn (M.B.); yaodong.he@tju.edu.cn (Y.H.); 2 Key Laboratory of Systems Bioengineering (Ministry of Education), Tianjin University, Tianjin 300072, China; 3 Qingdao Institute Ocean Engineering, Tianjin University, Qingdao 266237, China

**Keywords:** labyrinthulomycetes, marine, cultured thraustochytrids, elemental composition, carbon density, nitrogen density, biomass

## Abstract

The element stoichiometry of bacteria has received considerable attention because of their significant role in marine ecosystems. However, relatively little is known about the composition of major structural elements of the unicellular heterotrophic protists—thraustochytrids, despite their widely recognized contribution to marine nutrient cycling. Here, we analyze the cell volume and elemental C, N, H, and S cell content of seven cultured thraustochytrids, isolated from different marine habitats, in the exponential and stationary growth phases. We further derive the relationships between the cell volume and elemental C and N content of the cultured thraustochytrids. The cell volumes varied significantly (*p* < 0.001) among the isolates, with median values of 96.9 and 212.5 μm^3^ in the exponential and stationary phases, respectively. Our results showed a significantly higher percentage of C (64.0 to 67.5) and H (9.9 to 13.2) but a lower percentage of N (1.86 to 2.16) and S (0.34 to 0.91) in the stationary phase, along with marked variations of C and N fractions among isolates in the exponential phase. The cell C (5.7 to 203.7 pg) and N (0.65 to 6.1 pg) content exhibited a significant (*p* < 0.001) linear relationship with the cell volume (27.7 to 510 μm^3^). On further analysis of the relationship across the two growth phases, we found the equation (cell C (pg) = 0.356 × cell volume (μm^3^) + 20.922) for stationary phase cells more appropriate for C estimation of natural thraustochytrids. This study provides the first experimental evidence of higher cell C density than the current estimate and relatively larger C contribution of thraustochytrids than bacteria to the marine organic pool.

## 1. Introduction

Labyrinthulomycetes are widely distributed, saprotrophic, or only weakly parasitic fungus-like microorganisms, ubiquitous in estuarine and marine environments [1]. The morphologically described labyrinthulomycetes consist of a small group of almost exclusively marine genera—the thraustochytrids—with typically saprotrophic or bacteriotrophic, and occasionally holozoic nutrition [1,2]. Most thraustochytrids produce a fine ectoplasmic network of rhizoid-like threads that aid in anchoring to particulates and absorption of nutrients [3]. The presence of extensive ectoplasmic nets with highly degradative enzymes facilitates thraustochytrids in breaking down the complex, often recalcitrant organic matter [4]. Research over the last few decades has demonstrated that members of thraustochytrids thrive on dead autochthonous, as well as allochthonous, plant materials such as algal, mangrove, and seagrass detritus [2]. Thraustochytrids are also reported to occur in patches of very high density in the water column [5,6,7] and in fish farm-impacted seagrass sediments [8]. Further, their association with chlorophyll *a* and particulate organic C [6] and abundant presence in the oceanic waters [5] suggest their role in the degradation of autochthonous oceanic material. Apparently, thraustochytrids play an important role in the remineralization of complex organic materials in marine ecosystems. 

The biomass of thraustochytrids has previously been assessed indirectly in some studies using direct detection techniques [5,9,10,11,12,13,14] and flow cytometry [7,15]. Because of their high cell volume and C content, the biomass of thraustochytrids can approach that of bacteria in several ocean ecosystems [5,6,7,12,13]. In addition, they have been reported to occur at higher numbers than fungi in the water column [2] and were approximately equally abundant as fungi on the 0.2 μm filters [16]. The cells (10–20 μm average diameter) of thraustochytrids, which contain a large amount of polyunsaturated fatty acids (e.g., docosahexaenoic acid and eicosapentaenoic acid) with a high cholesterol concentration [17,18,19], are well within the preferred range of food particle size and can be grazed efficiently by zooplankton [8,20,21]. Inarguably, the large biomass contribution of thraustochytrids to microbes associated with particles and their serving as a food source for the zooplankton suggests their substantial role in marine C cycling and the food web [16,22].

Despite a growing body of evidence suggesting the significance of thraustochytrids in marine ecosystems, there is limited information regarding their elemental composition and biomass quantification. Similar to bacteria, the elemental composition of thraustochytrids determines the quality of the food material that is transferred to a higher trophic level via grazing [23]. The elemental composition also controls whether the particular microorganism excretes or consumes mineral nutrients [24]. Moreover, as microorganisms constitute the majority of living biomass in marine ecosystems, the knowledge about the contribution of particular organisms to the organic C pool is essential in understanding the trophic functioning of marine food webs [22,25].

In this study, we measured the major structural elements (C, N, H, and S) in cultured thraustochytrids by an elemental analyzer and determined the relationship between the cell biomass and volume. The objectives were to describe the variation of elemental composition during the growth of thraustochytrids under laboratory conditions and understand how the same growth medium affects the elemental content and elemental content-volume ratios of different isolates. This study also provides the statistical model to estimate the biomass C of natural thraustochytrids.

## 2. Results

### 2.1. Cell Mass and Volume

**Figure 1:**
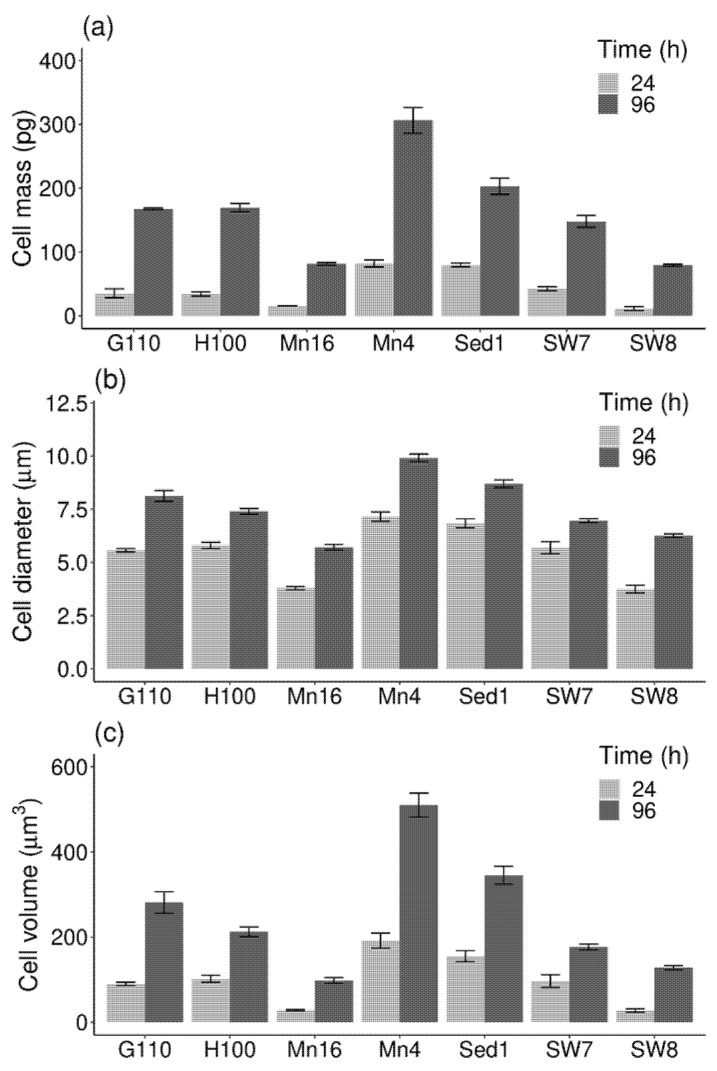
(**a**) Cell mass, (**b**) cell diameter, and (**c**) cell volume of different thraustochytrid isolates in the exponential (24 h) and stationary (96 h) phases of growth. Each bar represents the mean ± SD of triplicate samples.

The cell mass values of seven different thraustochytrid isolates ranged from 11.4 ± 3 (PKU#SW8) to 82.1 ± 5.5 pg (PKU#Mn4) in the exponential phase, and from 79.1 ± 1.7 (PKU#SW8) to 306.4 ± 20 pg (PKU#Mn4) in the stationary phase (Figure 1a). The values varied significantly (*p* < 0.001) among the isolates, with the median value of 35.2 ± 10.6 (median ± standard error) and 167.5 ± 29.3 pg in the exponential and stationary phases, respectively. Compared to the exponential phase cells, the stationary phase cells of all the isolates showed a marked increase in their dry weight mass.

The cell diameter of the exponential phase cells ranged from 3.75 ± 0.18 to 7.15 ± 0.22 μm while that of the stationary phase cells ranged from 5.72 ± 0.13 to 9.91 ± 0.18 μm (Figure 1b). All the isolates showed an increase in their cell diameter during growth. The cell volume of the isolates ranged from 27.7 ± 4.0 (PKU#SW8) to 191.7 ± 17.5 μm^3^ (PKU#Mn4) in the exponential phase, and from 97.9 ± 6.5 (PKU#Mn16) to 510 ± 27.9 μm^3^ (PKU#Mn4) in the stationary phase (Figure 1c). Similar to cell mass, the cell volumes varied significantly (*p* < 0.001) among the isolates, with the median values of 96.9 ± 22.8 and 212.5 ± 54 μm^3^ in the exponential and stationary phases, respectively. The stationary phase cells of all the isolates exhibited a marked increase in their volumes.

### 2.2. Elemental Composition

The cell C fraction (%) of the isolates ranged from 50.3 ± 1.02 (PKU#SW8) to 62.4 ± 1.42 (HNHK-100) in the exponential phase, and from 64.0 ± 0.20 (PKU#SW7) to 67.5 ± 0.90 (PKU#Sed1) in the stationary phase (Table 1). The variation of cell C fraction among the isolates in the exponential phase (coefficient of variation (CV) = 8.5%) was markedly higher than that in the stationary phase (CV = 2.1%). Between the exponential (median: 59.6% ± 1.9%) and stationary (median: 66.5% ± 0.5%) phases, the cell C fraction showed a significant (*p* < 0.01) increase. 

The cell N fraction (%) of the isolates ranged from 3.38 ± 0.06 (PKU#Sed1) to 6.73 ± 0.16 (PKU#Mn16) in the exponential phase, and from 1.86 ± 0.05 (PKU#SW7) to 2.16 ± 0.12 (GXBH-110) in the stationary phase (Table 1). The variation of cell N fraction among the isolates in the exponential phase (CV = 29.1%) was significantly higher than that in the stationary phase (CV = 6.3%), a pattern similar to that of the cell C fraction. However, in contrast to the cell C fraction pattern, the cell N fraction decreased (*p* < 0.001) between the exponential (median: 3.8% ± 0.49%) and stationary (median: 1.9% ± 0.05%) phases.

The cell H fraction (%) of the isolates ranged from 7.86 ± 0.12 (PKU#SW8) to 9.47 ± 0.19 (HNHK-100) in the exponential phase, and from 9.90 ± 0.34 (PKU#Mn4) to 13.24 ± 0.22 (GXBH-110) in the stationary phase (Table 1). The variation of cell H fraction among the isolates in the exponential phase (CV = 7.5%) was lower than that in the stationary phase (CV = 13.7%), which was in contrast to the cell C and N fractions pattern. Between the exponential (median: 9.0% ± 0.2%) and stationary (median: 11.23% ± 0.6%) phases, the cell H fraction showed a significant (*p* < 0.01) increase.

The cell S fraction (%) of the isolates ranged from 0.91 ± 0.03 (HNHK-100) to 1.82 ± 0.07 (PKU#SW8) in the exponential phase, and from 0.34 ± 0.01 (GXBH-110) to 0.91 ± 0.07 (PKU#SW8) in the stationary phase (Table 1). The variation of cell S fraction among the isolates in the exponential phase (CV = 26.7%) was lower than that in the stationary phase (CV = 40.0%), which was similar to the pattern of cell H fraction. Between the exponential (median: 1.09% ± 0.12%) and stationary (median: 0.44% ± 0.07%) phases, the cell S fraction showed a significant (*p* < 0.001) decrease.

**Figure 2:**
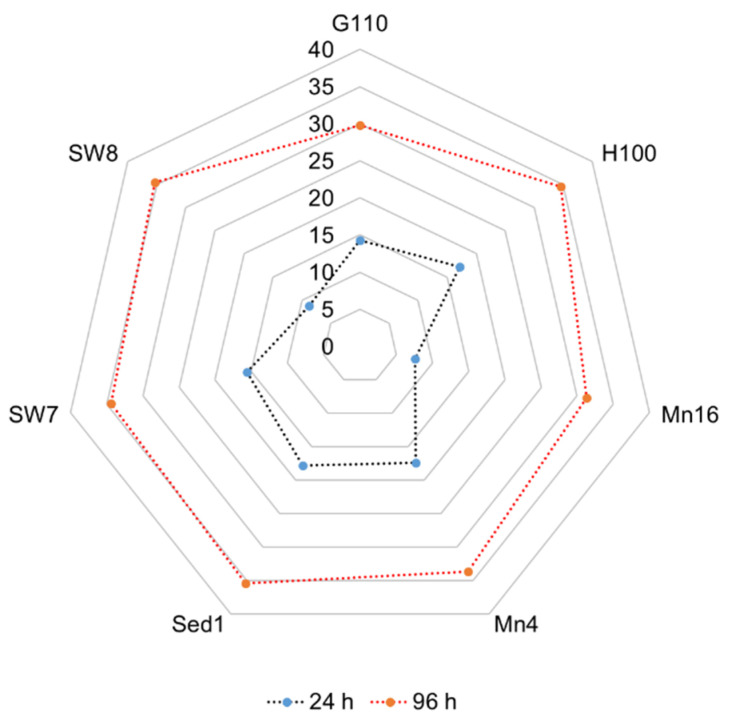
Changes in the C/N ratio of different thraustochytrid isolates during growth.

The C/N ratio of the isolates in their exponential and stationary phases varied within 7.61–17.85 (median: 15.7 ± 1.6) and 29.76–35.45 (median: 34.4 ± 0.8), respectively (Figure 2). The variation of the C/N ratio among different isolates in the exponential phase (CV = 29.9%) was higher than that in the stationary phase (CV = 6.4%). The C/N ratios of the isolates PKU#Mn16 and PKU#SW8 increased markedly from 7.61 to 31.36 and 8.81 to 35.35, respectively, during their growth.

### 2.3. Cell Carbon-to-Volume Relationship

**Figure 3:**
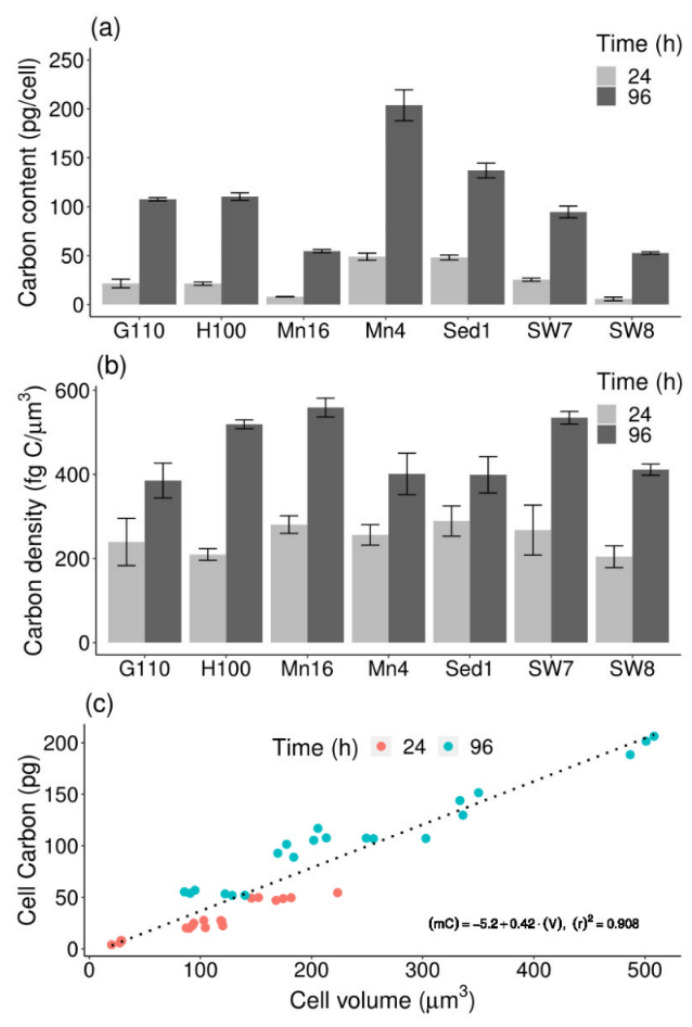
(**a**) Carbon content, (**b**) carbon density, and (**c**) relationship between carbon content and cell volume of different thraustochytrid isolates during growth. Each bar represents the mean ± SD of triplicate samples.

The C mass of isolates ranged from 5.7 ± 1.6 (PKU#SW8) to 48.9 ± 3.7 pg C/cell (PKU#Mn4) in the exponential phase, and from 52.6 ± 1.2 (PKU#SW8) to 203.7 ± 15.8 pg C/cell (PKU#Mn4) in the stationary phase (Figure 3a). The values of C mass varied significantly (*p* < 0.001) among the isolates, with the median values of 21.5 ± 6.5 and 107.6 ± 19.6 pg C/cell in the exponential and stationary phases, respectively. A significant increase in the C mass of all the isolates was observed during growth.

The cell C density (mass-to-volume ratio) ranged from 204 ± 26 (PKU#SW8) to 289 ± 36 fg C/μm^3^ (PKU#Sed1) in the exponential phase (median: 256 ± 13 fg C/μm^3^) and 385 ± 41 fg C/μm^3^ (PKU#Mn4) to 559 ± 22 fg C/μm^3^ (PKU#Mn16) in the stationary phase (median: 411 ± 29 fg C/μm^3^) (Figure 3b). The stationary phase cells of all the isolates showed significantly (*p* < 0.001) increased C densities compared with that of the exponential phase cells. In addition, the cell C densities varied significantly (*p* < 0.01) among the isolates in both the growth phases.

To determine the relationship between the C mass and volume of cultured thraustochytrid cells, a linear model was fitted to the experimental data. The result of model fitting showed a significant (*p* < 0.001) linear relationship between the C mass and volume (Figure 3c). Based on the linear model fit, the following regression equation was developed:

(1) $$m_{C}=0.42\times V-5.2\;\left(R^{2}=0.908\right)$$

where *m_C_* refers to cell C mass in pg and V stands for cell volume in μm^3^. 

### 2.4. Cell Nitrogen-to-Volume Relationship

**Figure 4:**
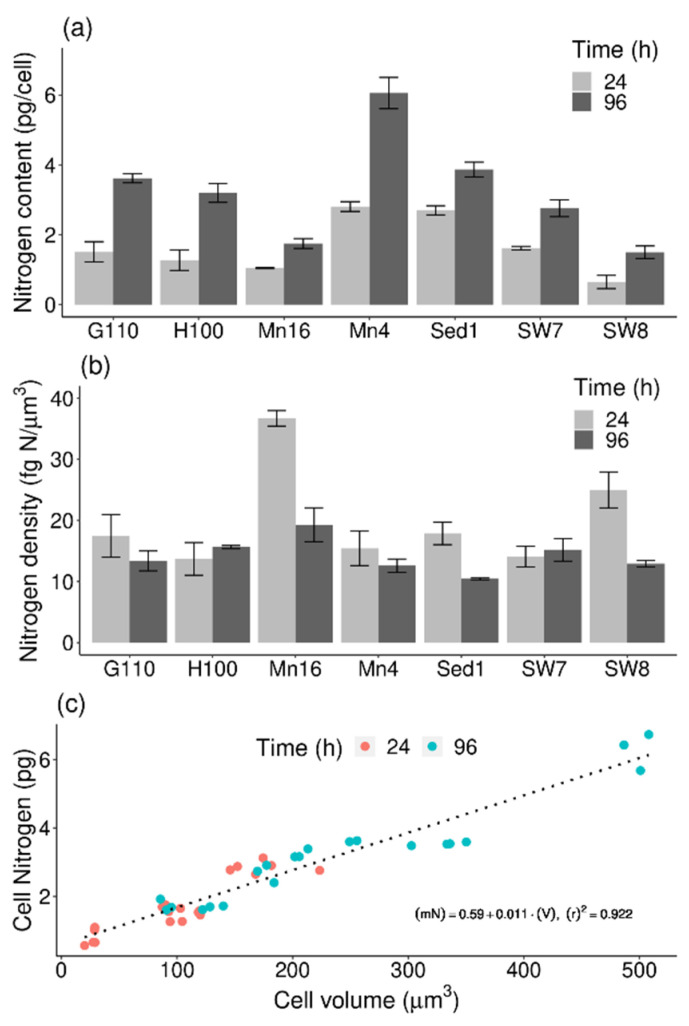
(**a**) Nitrogen content, (**b**) nitrogen density, and (**c**) relationship between nitrogen content and cell volume of different thraustochytrid isolates during growth. Each bar represents the mean ± SD of triplicate samples.

The N mass of isolates ranged from 0.65 ± 0.19 (PKU#SW8) to 2.8 ± 0.1 pg N/cell (PKU#Mn4) in the exponential phase, and from 1.5 ± 0.2 (PKU#SW8) to 6.1 ± 0.5 pg N/cell (PKU#Mn4) in the stationary phase (Figure 4a). The values varied significantly (*p* < 0.001) among the isolates, with the median values of 1.5 ± 0.3 and 3.2 ± 0.6 pg N/cell in the exponential and stationary phases, respectively. A significant increase in the N mass of stationary phase cells of all the isolates was observed.

The cell N density ranged from 13.7 ± 2.7 (HNHK-100) to 36.7 ± 0.1 fg N/μm^3^ (PKU#Mn16) in the exponential phase (median: 17.5 ± 3.1 fg N/μm^3^) and 10.5 ± 0.2 (PKU#Sed1) to 19.3 ± 2.8 fg N/μm^3^ (PKU#Mn16) in the stationary phase (median: 13.4 ± 1.1 fg N/μm^3^) (Figure 4b). The variation of cell N densities among the isolates was markedly higher in the exponential phase (CV = 41.3%) than that in the stationary phase (CV = 19.9%).

To determine the relationship between the N mass and volume of cultured thraustochytrid cells, a linear model was fitted to the experimental data. The result of model fitting showed a significant (*p* < 0.001) linear relationship between the N mass and volume (Figure 4c). Based on the linear model fit, the following regression equation was developed:

(2) $$m_{N}=0.011\times V+0.59\;\left(R^{2}=0.922\right)$$

where *m_N_* refers to N mass in pg/cell, and *V* stands for cell volume in μm^3^. 

## 3. Discussion

In this study, the cultured cells of all the thraustochytrid isolates were unicellular, globose to sub-globose (Figure S1), measuring 1.5 to 20 μm in diameter (Figures S2 and S3) during their growth on M4 medium. Similar cell morphology has been reported earlier for other isolated strains of thraustochytrids [12,26,27,28]. In natural seawater, the size of thraustochytrid cells was generally found to range in diameter from 5 to 20 μm [12]. The particle-bound cells of thraustochytrids were also reported to range from 3.5 to 19.7 μm [29]. Our results provide evidence that the cell diameter range of cultured thraustochytrids is comparable to that of the natural thraustochytrids. The cell volume of all the isolates increased more than two-fold between the exponential to the stationary phase. Generally, the exponential phase represents the period when most cells divide or produce microspores to proliferate, and hence the cells in this phase have a smaller average cell diameter than the stationary phase. Furthermore, the cell diameter and volume attributes exhibited wide differences among the isolates. As cultured thraustochytrids are known to produce intracellular lipids [30,31], the different cell sizes of the isolates perhaps suggest different levels of lipid accumulation within the cell. The factor(s) inducing differential levels of lipid content among various strains of thraustochytrids can be an interesting subject of future research.

C, N, S, P, and H are major elements that transfer metabolic energy and constitute the building blocks of a cell. These elements are required for the maintenance, growth, and reproduction of all living cells, and their determination is essential to quantify the flow of energy and nutrients among organisms in an environment [32,33]. Furthermore, microbial cells are known to be metabolically more active with rapid turnover of elemental contents and energy generation in the exponential phase of their growth than that in the stationary phase. In this study, the cellular contents of C, N, H, and S for different thraustochytrid isolates in their exponential and stationary phases of growth were measured and compared. The C:N:H:S ratios among the cultured thraustochytrids varied markedly during their growth on M4 medium (Table 1), suggesting different metabolic characteristics of the isolates. While the elements C and H tend to increase from the exponential to stationary phase of growth, the cell N and S decreased, which indicated a relative decline in the cellular protein content. Further, the high C/N ratios of isolates in the stationary phase indicated that the mature cells can have a significant contribution to the marine C pool. The C/N ratios of thraustochytrid cells (stationary phase) obtained in this study were about five to six times that of marine bacteria (coastal: 5.9 ± 1.1; oceanic: 6.8 ± 11.2) [34] and considerably higher than that (10.5 ± 1.1) of other previously reported cultured thraustochytrids [12]. Interestingly, the higher C content of thraustochytrids relative to the bacteria would relatively have a greater impact on the marine C cycle, highlighting the significance of thraustochytrids in marine ecosystems. In addition, the knowledge about the elemental content and their variations across growth phases acquired in this study would indeed benefit future research on the, yet to be understood, dynamics of elemental composition of natural thraustochytrids.

As mentioned above, the sizes of thraustochytrid cells vary widely in their natural habitats, ranging from 5 to 20 μm [12] or 3.5 to 19.7 μm [29]. The model equations for cell C and N estimations in this study are based on cell diameters that range from 3.7 μm to 9.9 μm; therefore, these models can provide a reliable estimate of the cell C or N content of natural thraustochytrid only within a cell diameter range of 3.5–10 μm. In the previous studies, the biomass C of natural thraustochytrids was estimated based on the abundance determined either by the use of a direct detection technique or MPN method. The measured abundance of natural thraustochytrids is then multiplied by the C content of a single cell to obtain the thraustochytrid biomass for the corresponding sample. The C content in most of the previous reports of thraustochytrid biomass was 2.05 × 10^−5^ μg/cell for a 5 μm diameter cell and 1.65 × 10^−4^ μg/cell for a 10 μm diameter cell as proposed by Kimura et al. [12]. The majority of the past studies, in particular, used the C content for a 10 μm diameter cell because natural thraustochytrid cells usually exhibit a larger cell diameter than 5 μm (geometric mean of 10 μm) [12]. The median cell C density (256 ± 13 fg C/μm^3^) of thraustochytrid isolates in the exponential phase of growth in this study was close to that (300 fg C/μm^3^) of cultured thraustochytrids reported previously [12]. However, for the stationary phase cells, which achieve the diameter comparable to that reported previously for natural thraustochytrids, the median cell C density (411 ± 29 fg C/μm^3^) was nearly 1.4-fold higher than the current estimate ((300 fg C/μm^3^), which suggests that thraustochytrids have a much larger contribution to the marine C pool than currently understood. Furthermore, it is interesting to note that the cell C densities varied significantly among the thraustochytrid isolates, which perhaps implies that different strains of thraustochytrids may not contribute equally to the marine C pool. This study provides the first line of evidence for such seemingly differential influence of thraustochytrid strains on marine C pool. 

**Figure 5:**
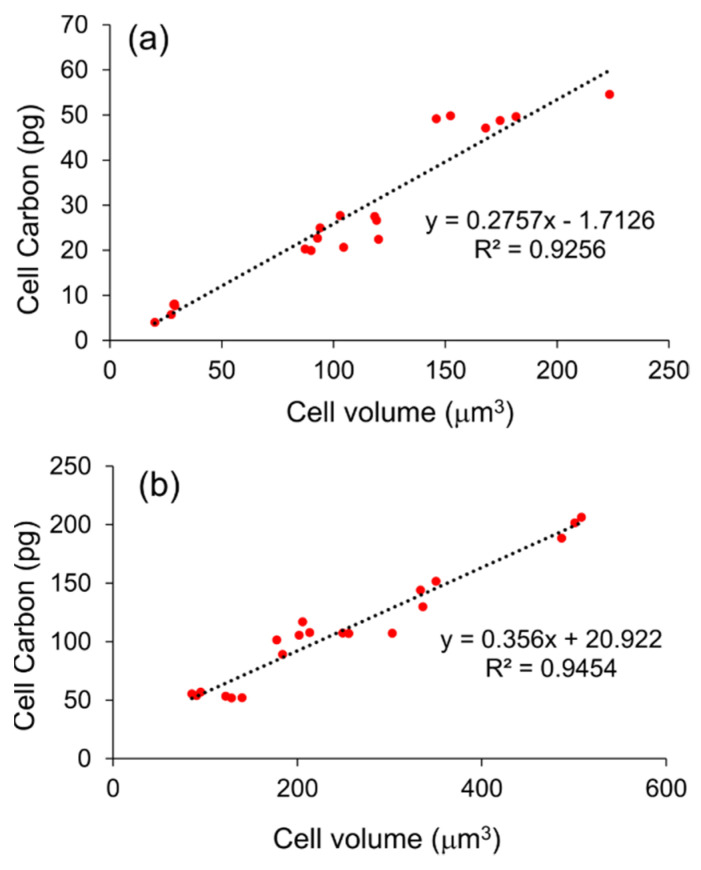
Linear regression of cell carbon content on cell volume of thraustochytrids during (**a**) the exponential phase, and (**b**) stationary phase of growth.

We observed that cells in the exponential phase of all isolates stained with acriflavine revealed only green fluorescing nuclei, while cells in the stationary phase revealed distinct orange-to-red fluorescing cell walls and yellow-to-green fluorescing nuclei (Figure S1). The orange-to-red fluorescing cell walls have been attributed to the presence of sulfated polysaccharides, and micrographs of natural thraustochytrids mostly show orange-to-red fluorescing cell walls [29]. Moreover, the majority of the microorganisms in the natural habitats are predominantly present in the stationary phase [35]. As natural habitat often contains limited nutrients, rapid growth is usually hampered. Furthermore, there are other conditions, including physical and chemical stresses, which result in unbalanced growth [36]. Taking the above into consideration, we derived two linear regression equations, each for the exponential (cell C (pg) = 0.2757 × cell volume (μm^3^) − 1.7126) and stationary (cell C (pg) = 0.356 × cell volume (μm^3^) + 20.922) growth phases (Figure 5) to obtain a more accurate model for estimation of cell C of natural thraustochytrids based on cell volume measurements. Our study suggests that it would be appropriate to use the equation derived for the stationary phase cells while estimating the biomass C of natural thraustochytrids. However, the biomass C of natural thraustochytrid populations may vary considerably with the trophic status of their environment. Our proposed conversion factor, which is derived based on thraustochytrids cultured under nutrient-replete conditions, would provide more accurate estimates of biomass C of natural thraustochytrids from environments under eutrophic conditions. More importantly, the application of our experimentally determined conversion factor will aid in quantifying the potential contribution of thraustochytrids to the global microbial biomass. Nevertheless, until a method to directly measure the biomass C of natural thraustochytrids is developed, we consider that our model equation would greatly benefit researchers interested in studying the biology and ecology of natural thraustochytrids. 

## 4. Materials and Methods

### 4.1. Isolates and Culture Conditions

Seven thraustochytrid isolates (Table 2), previously isolated from three different marine habitats [30,31], were used in the present study. The isolates were maintained at 28 °C on modified Vishniac’s (MV) medium (glucose 10 g/L, peptone 1.5 g/L, yeast extract 0.1 g/L, 100% artificial seawater, and agar 20 g/L) and subcultured every 25 days, as described in our previous studies [30,31]. The seed culture of each isolate was prepared separately by inoculating a loopful of cells from the agar plate into a flask (100 mL) containing 50 mL of the M4 medium (glucose, 20 g/L; peptone, 1.5 g/L; yeast extract, 1 g/L; KH2PO4, 0.25 g/L; and 100% artificial seawater, pH = 7) and then incubating the culture flask at 28 °C for 24 h under reciprocal shaking (170 rpm). The resulting seed culture (5% v/v) was then transferred to a 100 mL shake flask containing 40 mL fresh M4 medium and cultivated on an orbital shaker for 3 days under the same conditions.

### 4.2. Analysis of Elemental Composition

The elemental composition (C, N, H, and S) of the biomass of thraustochytrid isolates was analyzed for the exponential (24 h) and stationary (96 h) growth phases. The time points 24 h and 96 h were chosen as exponential and stationary phases, respectively, based on the growth curves of the isolates (Figure S4). The isolates were cultivated in 50 mL of sterilized M4 medium inoculated with respective seed cultures (5% v/v) for 7 days at 28 ℃ and 170 rpm. Seed cultures (5% v/v) were prepared as described in Section 4.1. Samples were collected from the culture broth and a subsample of about 8 mL was used for cell harvesting by centrifugation (20 min, 8000 rpm, 20 ℃). The resulting cell pellet was washed several times with 5 mL of sterile deionized water and pre-frozen at −80 ℃ and then lyophilized in a freeze dryer (CHRIST ALPHA 1-2 LD Plus, Germany) for 48 h. The freeze-dried cells were weighed to obtain their dry cell weight. The elemental composition of the freeze-dried cells (2 mg) was determined on a vario MACRO cube elemental analyzer (Elementar Analysensysteme GmbH, Germany) following the manufacturer’s instructions. 

### 4.3. Determination of Cell Count, Diameter, and Volume 

Thraustochytrid cells in the culture sample were enumerated by the direct detection technique described in our previous study [13]. About 30–40 microscopic fields were scanned with blue light (450–490 nm) excitation under a fluorescence microscope (Olympus BX53, Olympus Corporation, Tokyo, Japan), and the number of observed cells was recorded. The cell diameter was measured using the ImageJ software (https://imagej.net/, accessed on 21 August 2021). Based on the cell radius (*r*) and globose to subglobose shape of cells, the cell volume (*V*) was calculated by using the formula

(3) $$V=\;\frac{4}{3}{\pi r}^{3}$$

### 4.4. Statistical Analyses

The mean, median, and standard error for each measured parameter and the test of significance (ANOVA) were computed in R software (version 4.0.0, https://www.r-project.org, accessed on 21 August 2021). Prior to performing ANOVA, the homogeneity of variance was tested using Levene’s test of the R car package. Linear regression analysis was performed using the R stats package. Data were plotted using the R ggpplot2 package and Microsoft Excel. 

## Figures and Tables

**Table 1:** Elemental composition of different thraustochytrid isolates in exponential and stationary phases of growth.

Growth Phase	Isolate	C (%)	N (%)	H (%)	S (%)	C:N:H:S
Exponential (24 h)	G110	61.10 ± 0.81	4.29 ± 0.05	9.28 ± 0.09	1.00 ± 0.02	61:4:9:1
	H100	62.43 ± 1.42	3.69 ± 0.50	9.47 ± 0.19	0.91 ± 0.03	62:4:9:1
	Mn16	51.23 ± 0.17	6.73 ± 0.16	7.90 ± 0.03	1.21 ± 0.09	51:7:8:1
	Mn4	59.56 ± 0.44	3.42 ± 0.06	8.57 ± 0.52	0.95 ± 0.19	60:3:9:1
	Sed1	60.39 ± 0.05	3.38 ± 0.06	9.13 ± 0.01	1.10 ± 0.05	60:3:9:1
	SW7	59.64 ± 1.47	3.82 ± 0.24	9.01 ± 0.20	1.09 ± 0.00	60:4:9:1
	SW8	50.27 ± 1.02	5.71 ± 0.16	7.86 ± 0.12	1.82 ± 0.07	50:6:8:2
Stationary (96 h)	G110	64.28 ± 0.43	2.16 ± 0.10	13.90 ±0.22	0.34 ± 0.01	640:20:140:3
	H100	65.05 ± 0.27	1.89 ± 0.10	13.24 ±0.22	0.36 ± 0.01	325:10:65:2
	Mn16	66.94 ± 1.07	2.14 ± 0.12	10.57 ±0.22	0.46 ± 0.01	134:4:22:1
	Mn4	66.46 ± 0.92	1.98 ± 0.05	9.90 ±0.34	0.49 ± 0.01	132:4:20:1
	Sed1	67.50 ± 0.90	1.91 ± 0.08	11.23 ±0.22	0.44 ± 0.01	340:10:55:2
	SW7	64.01 ± 0.20	1.86 ± 0.05	11.90 ±0.22	0.41 ± 0.01	160:5:30:1
	SW8	66.57 ± 0.65	1.90 ± 0.24	9.93 ±0.07	0.91 ± 0.07	670:20:100:9

Note: Each value represents the mean ± SD of triplicate samples.

**Table 2:** Thraustochytrid isolates used in this study.

Isolate	Isolation Source	GenBank Accession	% Homology with Type Strain ^#^
GXBH-110 (G110)	Mangrove leaves	MG429124.1	99.56
HNHK-100 (H100)	Mangrove leaves	MG429118.1	99.18
PKU#Mn16 (Mn16)	Mangrove leaves	JX847368.1	97.83
PKU#Mn4 (Mn4)	Mangrove leaves	JX847360.1	98.80
PKU#Sed1 (Sed1)	Sediment	JX847370.1	98.86
PKU#SW7 (SW7)	Seawater	JX847377.1	98.75
PKU#SW8 (SW8)	Seawater	JX847378.1	98.70

# *Aurantiochytrium limacinum* ATCC MYA-1381 (GenBank: AB973564.1).

## Supplementary Materials

The following are available online at https://www.mdpi.com/article/10.3390/md19090493/s1, Figure S1: Micrographs of acriflavine-stained thraustochytrid cells. (a) PKU#Mn4 cells at 24 h, (b) PKU#Mn4 cells at 96 h, (c) PKU#Mn16 cells at 24 h, and (d) PKU#Mn16 cells at 96 h. Figure S2: Cell size distribution of thraustochytrid isolates in exponential phase of growth. Figure S3: Cell size distribution of thraustochytrid isolates in stationary phase of growth. Figure S4: Growth curves of thraustochytrid isolates used in this study.
