# Supplementary material for: Elemental Composition and Cell Mass Quantification of Cultured Thraustochytrids Unveil Their Large Contribution to Marine Carbon Pool
Source: Mar Drugs. 2021 Aug 29;19(9):493. doi: 10.3390/md19090493 (PMC8468426; doi:10.3390/md19090493)
Supplement: Supplementary file 1 [file marinedrugs-19-00493-s001.zip › marinedrugs-1343612-supplementary.pdf]

# Supplementary Information

## Elemental Composition and Cell Mass Quantification of Cultured Thraustochytrids Unveil their Large Contribution to Marine Carbon Pool

**Biswarup Sen [1], Jiaqian Li [1], Lyu Lu [1], Mohan Bai [1], Yaodong He [1] and Guangyi Wang [1,2,3,*]**

[1] Center for Marine Environmental Ecology, School of Environmental Science and Engineering, Tianjin University, Tianjin 300072, China; bsen@tju.edu.cn (B.S.); lijiaqian@tju.edu.cn (J.L.); lvluu@tju.edu.cn (L.L.), bmh@zju.edu.cn (M.B.); yaodong.he@tju.edu.cn (Y.H.); gywang@tju.edu.cn (G.W.)

[2] Key Laboratory of Systems Bioengineering (Ministry of Education), Tianjin University, Tianjin 300072, China

[3] Qingdao Institute Ocean Engineering, Tianjin University, Qingdao 266237, China

* Correspondence: gywang@tju.edu.cn; Tel: (86) 022–8740210

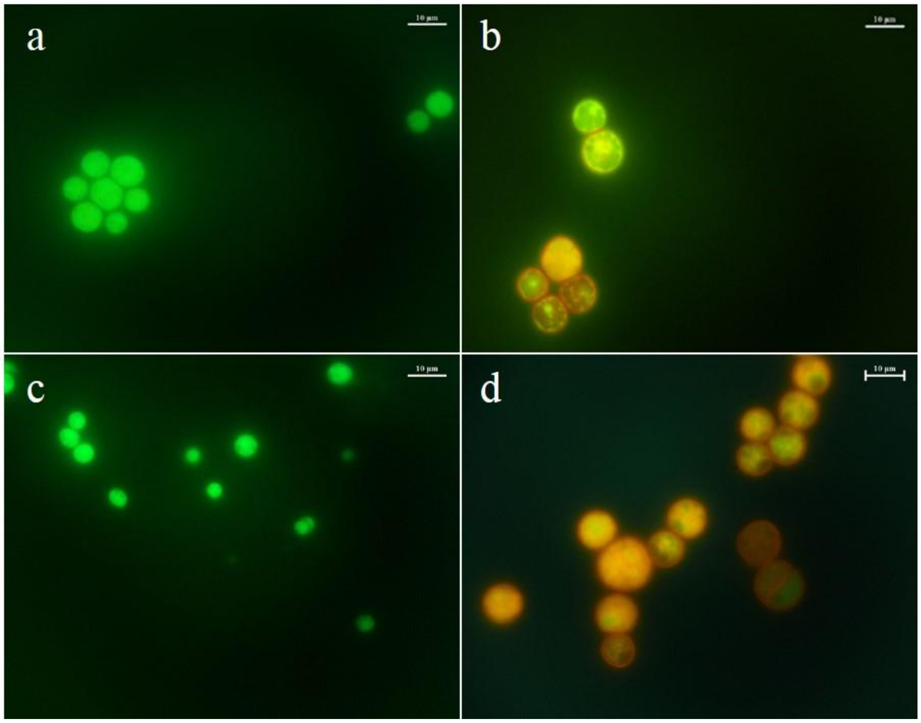


**Figure S1**: Micrographs of acriflavine-stained thraustochytrid cells. (a) PKU#Mn4 cells at 24 h, (b) PKU#Mn4 cells at 96 h, (c) PKU#Mn16 cells at 24 h, and (d) PKU#Mn16 cells at 96 h.

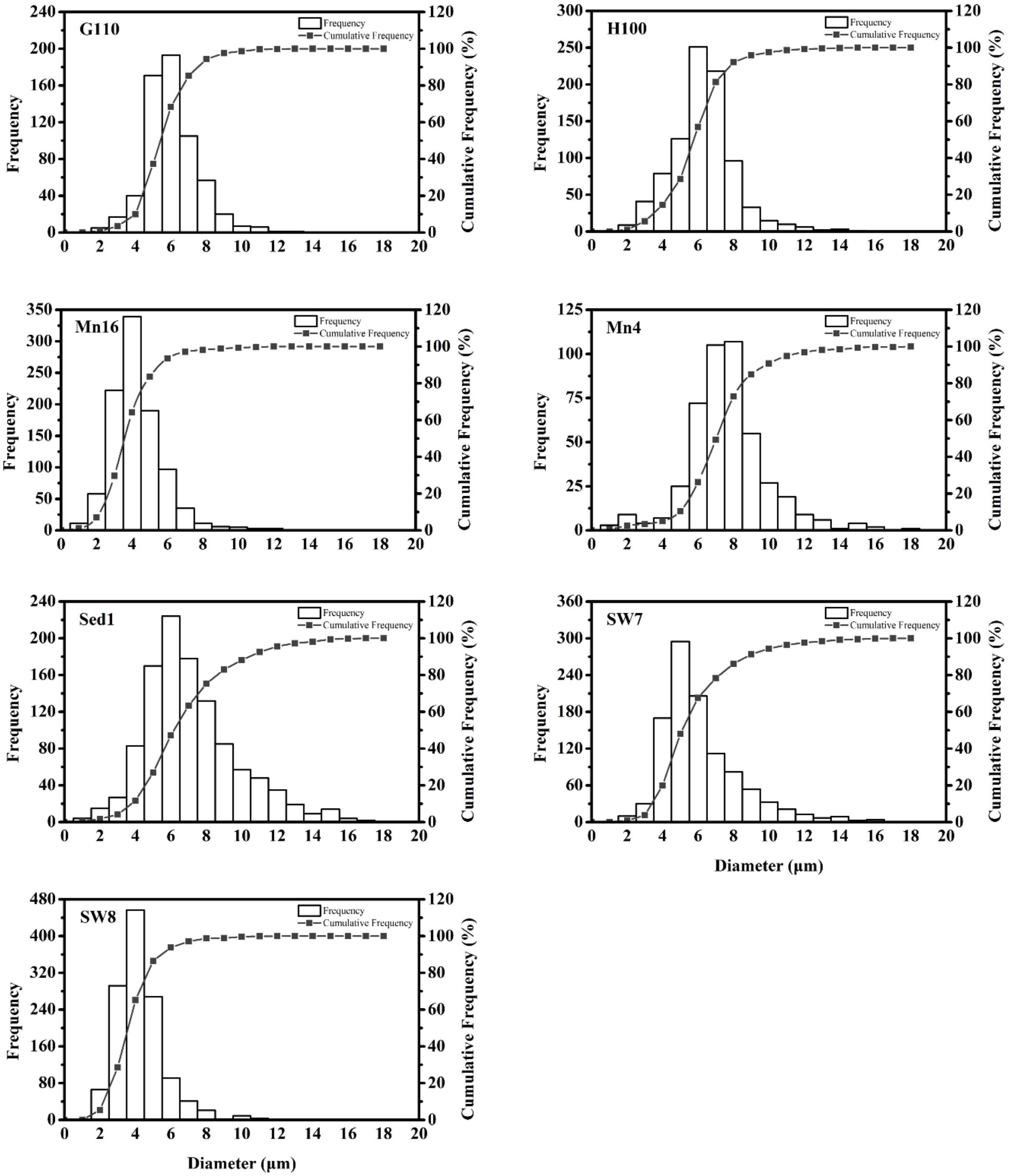


**Figure S2**: Cell size distribution of thraustochytrid isolates in exponential phase of growth.

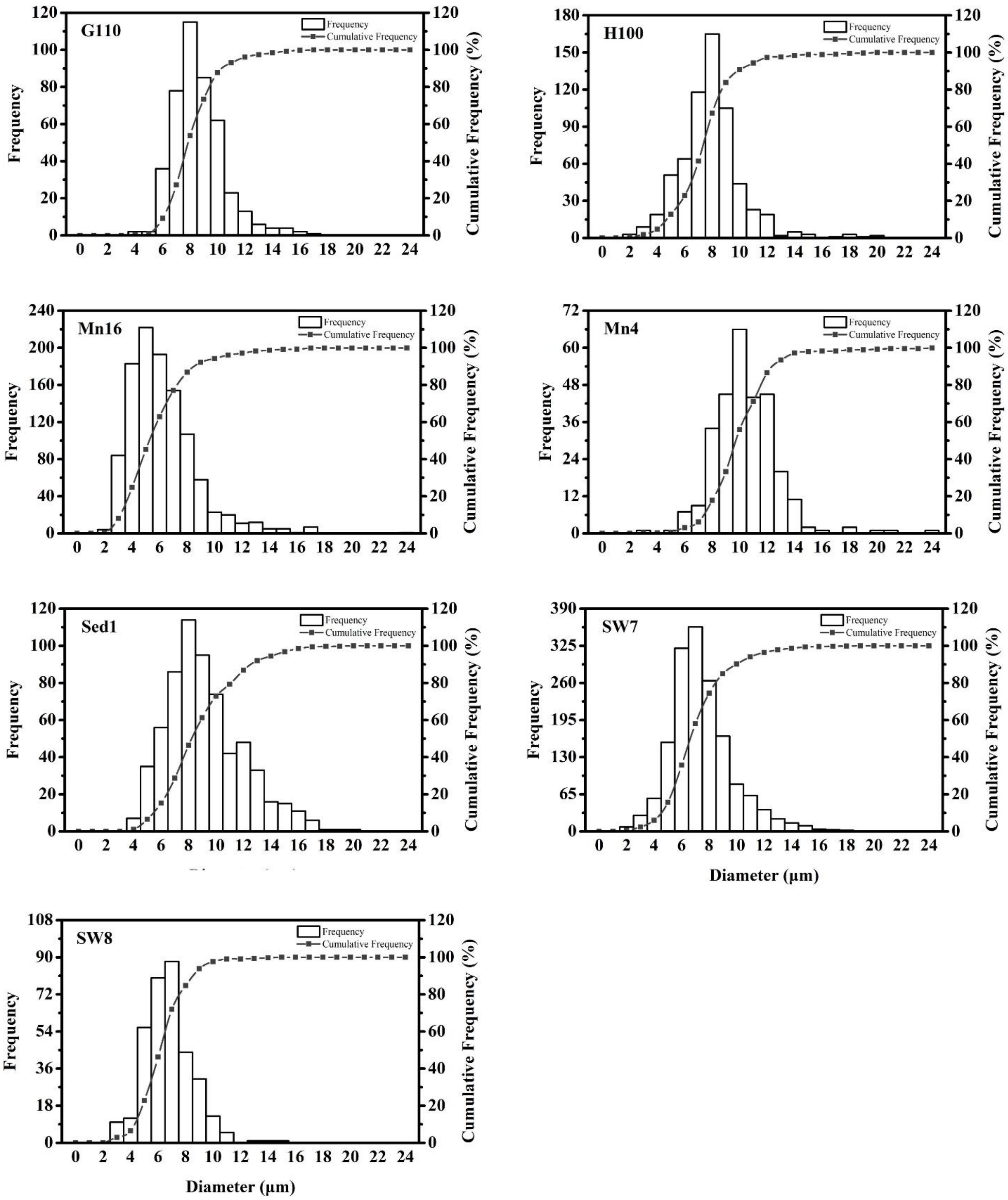


**Figure S3**: Cell size distribution of thraustochytrid isolates in stationary phase of growth.

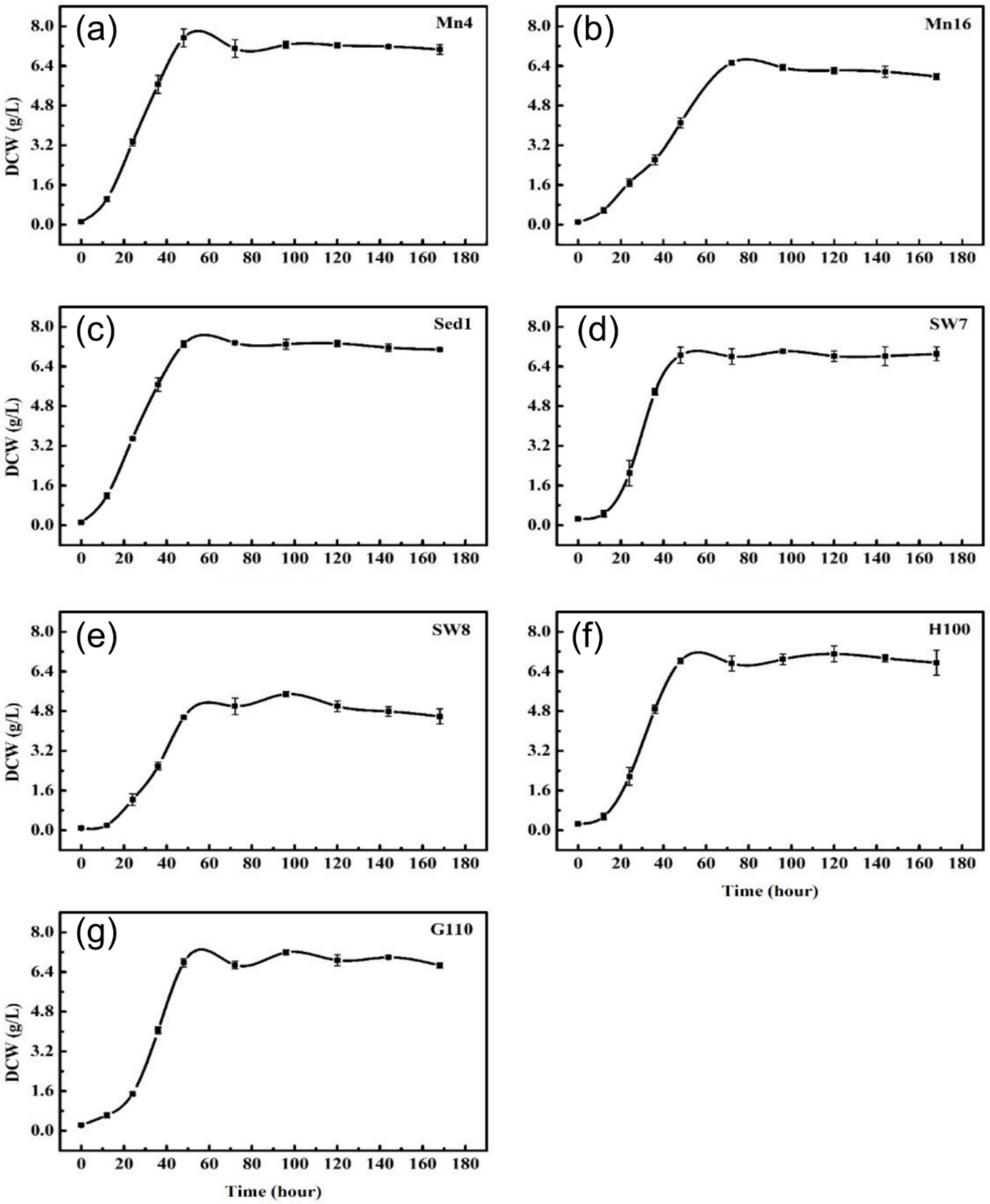


**Figure S4:** Growth curves of thraustochytrid isolates used in this study.
